# Satisfaction evaluation of flash glucose monitoring system in early glucose management of patients after kidney transplantation

**DOI:** 10.3389/fmed.2025.1557599

**Published:** 2025-10-08

**Authors:** Jiayu Guo, Ming Qin, Jinke Li, Jiangqiao Zhou, Tao Qiu, Yan Tang

**Affiliations:** ^1^Department of Organ Transplantation, Renmin Hospital of Wuhan University, Wuhan, Hubei, China; ^2^Department of Urology, Renmin Hospital of Wuhan University, Wuhan, Hubei, China

**Keywords:** renal transplantation, blood glucose monitoring, the flash glucose monitoring system, glucose abnormalities, advantage

## Abstract

**Aim:**

To study the effectiveness of the flash glucose monitoring (FGM) system in the monitoring of blood glucose in patients after renal transplantation.

**Methods:**

One hundred and fifteen patients who underwent renal transplantation at the Renmin Hospital of Wuhan University from January to December 2021 were selected for the study, with patients from January to June as the control group (*n* = 62) and patients from July to December as the observation group (*n* = 53). The control group used traditional finger blood collection to monitor blood glucose, while the observation group used FGM system to monitor the patients' blood glucose. The Digital Pain Rating Scale (NRS) and the Glucose Monitoring System Satisfaction Questionnaire (GMSS) were used to compare the pain associated with glucose needling and patient satisfaction with the glucose monitoring equipment, and compared the incidence of abnormal blood glucose events and adverse events between the two groups.

**Results:**

The differences in pain comparison, satisfaction with the blood glucose monitoring equipment, the number of abnormal blood glucose events and adverse events between the two groups were statistically significant (*p* < 0.05).

**Conclusion:**

The application of FGM system enables continuous glucose monitoring and management of patients in the early post-transplant period, reduces the painful pinprick of glucose monitoring, detects glucose abnormalities early, reduces adverse events and improves patient satisfaction.

## 1 Introduction

Renal transplantation is the best treatment for patients with end stage renal disease (ESRD) and can significantly improve survival and quality of life. Patients need to take immunosuppressive drugs for life after renal transplantation. However, almost all immunosuppressive drugs have been shown to produce metabolism-related adverse effects, and surgical stimulation and rehydration can also increase metabolic abnormalities, with glucose abnormalities being the most common (1–4).

The studies have shown that early hyperglycemia occurs in up to 75–92% of kidney transplant patients within 1 week of transplantation (5, 6) with 29–87% having at least one episode of hyperglycemia and 3.7% having at least one episode of hypoglycemia after surgery (6–8). Fasting hyperglycemia occurring in the first week after transplantation has been shown to be the strongest predictor of post-transplantation diabetes mellitus (PTDM) 1 year later (9). PTDM has been identified as the second most important factor affecting long-term patient survival apart from acute and chronic rejection (10, 11) and is also the most important factor affecting transplant rejection (12) 75% of patients who developed hyperglycemia in the first week after renal transplantation will develop diabetes during the 3-year follow-up period (13) More recent evidence further underscores the significance of early post-transplant dysglycemia. A comprehensive review by Iqbal et al. (14) highlighted that early hyperglycemia is not merely a transient phenomenon but a critical mediator of adverse outcomes, including an increased risk of infections and impaired graft function, necessitating vigilant monitoring and management (15) Moreover, the latest expert consensus on Post-Transplant Diabetes Mellitus (PTDM) management emphasizes the importance of early glycemic monitoring and acknowledges the potential role of continuous glucose monitoring (CGM) technologies in high-risk populations during the immediate post-operative period (16). At the same time, post-transplant hypoglycemia is an important risk factor for falls, and severe hypoglycemia can lead to coma and even death (17, 18). Therefore, monitoring of post-transplant glucose is essential for the recovery of transplanted kidney function and the long-term survival of the kidney transplant recipient.

Considering the high impact of surgical stress, high-dose hormone shock in a short period of time and the application of immunosuppressive drugs on blood glucose fluctuations, current studies about blood glucose management in renal transplant patients focus on patients who are stable (>3 months) or more after renal transplantation, and are mostly diagnosed diabetic patients. There are fewer studies on blood glucose monitoring and blood glucose management in the early post-transplant period. However, the damage to the kidneys from fluctuations in blood glucose may be more severe than from persistent hyperglycemia (19). We need a better way to monitor blood glucose in the early stages after kidney transplantation to detect abnormal blood glucose in post-transplant patients early, thus avoiding blood glucose fluctuations and reducing the incidence of diabetes. The most commonly used blood glucose monitoring method in clinical practice is capillary blood glucose monitoring. However, due to its frequent invasive operation, patients will suffer pain, and irregular operation may also affect the accuracy of blood glucose monitoring level, seriously affecting patients' quality of life and enthusiasm and compliance of blood glucose monitoring. Continuous glucose monitoring (CGM) is a technology that continuously monitors glucose concentration in subcutaneous interstitial fluid through glucose sensors, which can provide continuous, comprehensive and reliable whole-day blood glucose information. Clinical studies at home and abroad have proved that CGM has good accuracy and safety (20, 21).

Flash glucose monitoring (FGM) is a form of continuous glucose monitoring (CGM) that requires the user to actively scan the sensor with a reader to obtain current glucose readings and trend data (22). Unlike CGM, which transmits data automatically, FGM provides intermittent but comprehensive glucose profiles with significantly reduced fingerstick calibrations (23). In this study, we used the FreeStyle Libre FGM system, which patients scanned intermittently to obtain glucose values.

Flash glucose monitoring (FGM) is a new, safe and less invasive method of continuous glucose monitoring and has been shown to be more accurate when used in post-operative glucose monitoring in renal transplant patients (20). Therefore, in this study, FGM was used for glucose monitoring in patients in the early post-operative period after renal transplantation thereby observing the level of glucose fluctuation in patients and its value of application in clinical practice.

## 2 Methods

### 2.1 Study subjects

One hundred and fifteen kidney transplant recipients who underwent renal transplantation in Renmin Hospital of Wuhan University from January to December 2021 were selected as study subjects. All study subjects were divided into a control group (*n* = 62) and an observation group (*n* = 53) the chronological order of admission. The post-operative immunosuppressive regimen for all patients was tacrolimus (TAC) + mycophenolate (MMF) + prednisone.

Inclusion criterias are: (1) age > 18 years; (2) patients receiving their first kidney transplant; (3) wear FGM on the first day after surgery and use it for up to 14 days; (4) Patients in the control group performed capillary blood glucose monitoring at least four times daily (fasting and 2 hours after each meal); and (5) patients sign an informed consent form and have good compliance.

Exclusion criterias are: (1) multi-organ combination transplant; (2) Pre-operative use of glucocorticoids for more than 3 months; (3) allergy to compresses; and (4) extremely thin or severe edema.

All patients in this study signed an informed consent form and the study protocol was conducted in accordance with the Declaration of Helsinki. The study was approved by the Ethics Committee of Renmin Hospital of Wuhan University.

### 2.2 Patient clinical information and assessment

The clinical data of the 115 patients, such as sex, age, Body Mass Index (BMI), family history of diabetes mellitus, dialysis history, primary disease, donor kidney source, history of hepatitis C, number of comorbidities, fasting plasma glucose (FPG), 2 h post-prandial blood glucose (2hPG), pre-operative glycated hemoglobin (HbA1c), pain levels, glucose monitoring system satisfaction, abnormal blood sugar events, and adverse events were all collected.

In the control group, blood glucose monitoring was performed using Roche conventional finger blood collection (ACCUCHEK^®^ Performa, Roche, Shanghai, China) and in the observation group, blood glucose monitoring was performed using FGM (FreeStyle Liber HAbbott, Shanghai, China). Blood glucose monitoring was performed from the day of surgery to 15 days after surgery, on a fasting basis and 2 h after three meals. Patients should be monitored at any time in case of abnormal blood glucose. All patients had pre-operative glycated hemoglobin measured by venous blood, with normal values ranging from 3.6 to 6.0% and a diagnosis of diabetes when ≥6.5%.

Patients' pain levels were evaluated according to numerical rating scale (NRS) (24). The scale was completed by the investigator on the 3rd, 7^th^, and 15th post-operative days, with the patient being instructed to fill in the scale according to their real situation.

Patient satisfaction with the glucose monitoring device was surveyed using the Glucose Monitoring System Satisfaction Survey (GMSS) (25). The questionnaire was completed by the investigator on the 15th post-operative day after the patient was instructed to complete it according to the patient's real situation.

The American Diabetes Association guidelines (26) for abnormal blood sugar were used to define abnormal blood glucose events. Hypoglycemia is defined when blood sugar is ≤ 3.9 mmol/L or 70 mg/dL, and hyperglycemia is defined when blood sugar is ≥10 mmol/L or 180 mg/dL. The occurrence of adverse events was mainly recorded according to the main complaint or actual occurrence of patients, including falls, polydipsia, wound infection, etc.

All patients with abnormal glucose events received standardized clinical interventions according to the institutional protocol. Hypoglycemia ( ≤ 3.9 mmol/L) was treated immediately with oral carbohydrates or intravenous glucose infusion. Hyperglycemia (≥10.0 mmol/L) was managed with subcutaneous or intravenous insulin therapy under physician guidance, following a sliding scale protocol.

### 2.3 Statistical analysis

SPSS 26.0 software was used for statistical analysis. Quantitative data with a normal distribution were expressed as the mean ± standard deviation (SD), and a *t*-test was used to compare the data of two groups. Quantitative data that do not conform to a normal distribution were expressed as median, and non-parametric test was used to compare the data of two groups. Qualitative data were compared using a χ^2^ test. *P* < 0.05 was considered statistically significant. A *post-hoc* power analysis was performed using G^*^Power software. With the observed effect sizes for the primary outcomes (e.g., satisfaction scores, pain scores), the achieved statistical power exceeded 80% at a significance level of α = 0.05, indicating that the sample size was adequate to detect significant differences between the groups.

## 3 Results

### 3.1 Basic characteristics of two patient groups

**Figure 1:**
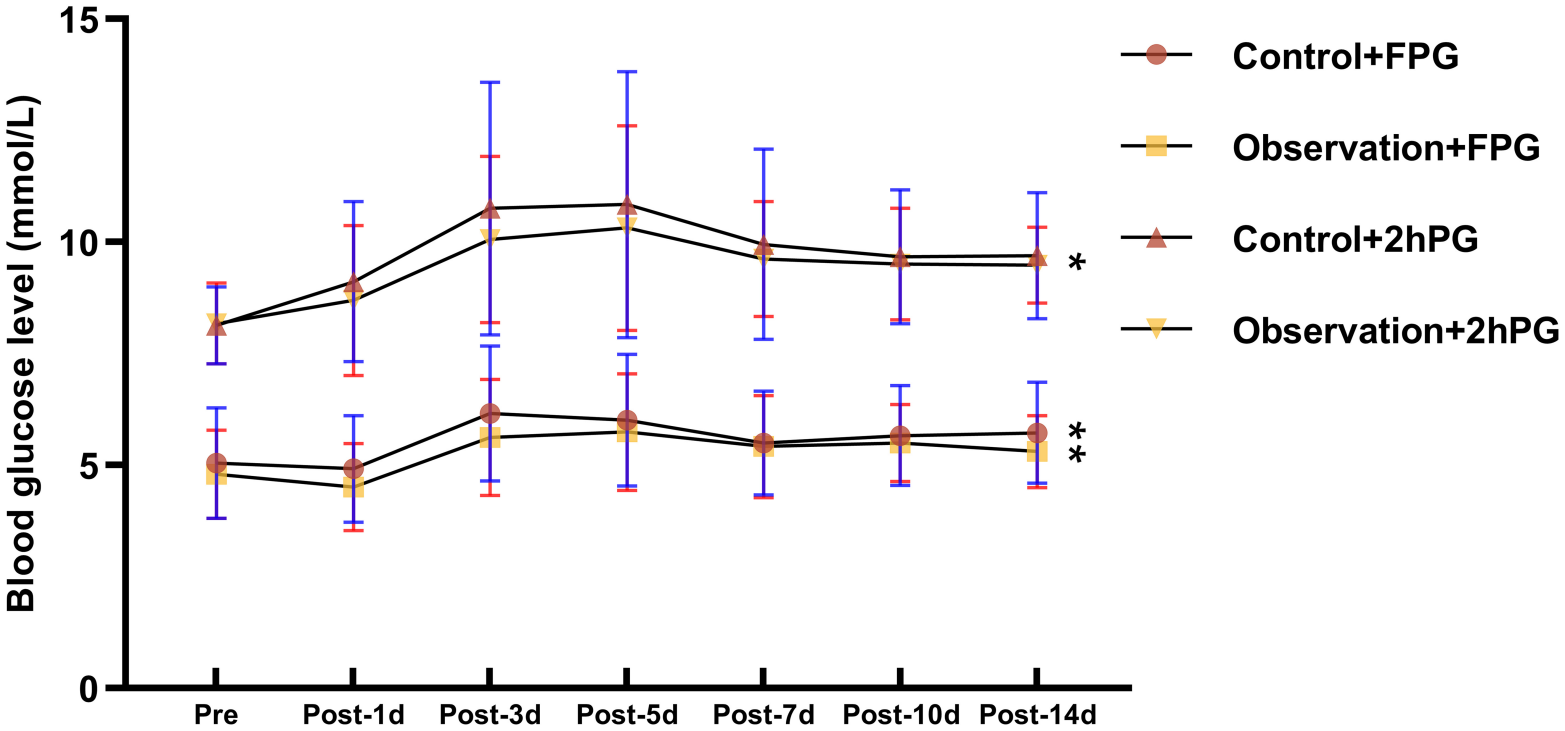
Comparison of fasting glucose and 2-h post-prandial glucose before and after renal transplantation. FPG, fasting plasma glucose; 2HPG, 2 h post-prandial blood glucose. * represents: <0.05; **represents: <0.01

This study included a total of 115 patients (57 males and 58 females) with ages ranging from 20 to 60(average: 40.93 ± 11.06) years. All patients were first-time kidney transplant recipients and were on an immunosuppressive regimen of TAC + MMF + prednisone after surgery. Fifteen of all patients had a family history of diabetes, and only 11 patients did not suffer from dialysis treatment prior to surgery. Most of recipients received donor kidneys from organ donation after the donor had undergone cardiac death. No significant differences in sex, age, BMI, family history of diabetes mellitus, dialysis history, primary disease, donor kidney source, history of hepatitis C, number of comorbidities, FPG, 2hPG, and pre-operative HbA1c between the two groups were found (Table 1). However, all patients had higher blood glucose after kidney transplantation than before surgery, and all had lower blood glucose than the control group using FPG monitoring (Figure 1).

**Table 1:** Basic characteristics all study patients.

**Group**	**Total (*n* = 115)**	**Control (*n* = 62)**	**Observation (*n* = 53)**	***t*/c^2^**	***P-*value**
**Sex (** ***n*** **)**					
Male	57	28	29	0.696	0.404
Female	58	34	24
Mean age (year)	40.93 ± 11.06	40.8 ± 11.63	40.98 ± 10.45	0.045	0.964
BMI	23.08 ± 2.36	23.03 ± 2.47	23.13 ± 2.25	0.236	0.814
**History of diabetes (** ***n*** **)**					
None	100	54	46	0.000	1.000
Exist	15	8	7		
**Protopathy (** ***n*** **)**					
IgA nephropathy	18	9	9	2.581	0.859
Hypertensive nephropathy	18	10	8		
Polycystic kidney	3	2	1		
Primary glomerulonephritis	33	16	17		
Lupus nephritis	12	7	5		
Diabetic nephropathy	11	8	3		
None	20	10	10		
**Dialysis**					
None	11	4	7	2.093	0.351
Yes	104	58	46		
**Donor kidney source**					
DCD	107	57	50	0.019	0.891
Dindred	8	5	3		
**History of hepatitis C**					
None	110	59	51	0.000	1.000
Yes	5	3	2		
**Number of comorbidities (** ***n*** **)**					
0	18	12	6	3.345	0.341
1	60	30	30		
2	35	18	17		
3	2	2	0		
Pre-FPG (mmol/L)	4.93 ± 1.13	5.04 ± 1.23	4.80 ± 0.99	1.172	0.243
Pre-2HPG (mmol/L)	8.15 ± 0.88	8.13 ± 0.86	8.17 ± 0.94	0.249	0.803
Pre-HbA1c (%)	6.02 ± 0.89	6.01 ± 0.97	6.02 ± 0.79	0.037	0.970

DCD, Donation after cardiac death; Pre-FPG, pre-operation fasting plasma glucose; Pre-2HPG, pre-operation 2 h post-prandial blood glucose; Pre-HbA1c, pre-operation glycated hemoglobin; BMI, body mass index.

Data are presented as mean ± standard deviation, n (number), or median as appropriate. Comparisons were made using Student's *t*-test, Chi-square test, or Mann–Whitney *U* test.

### 3.2 Comparison of pain during blood glucose monitoring between the two groups

**Figure 2:**
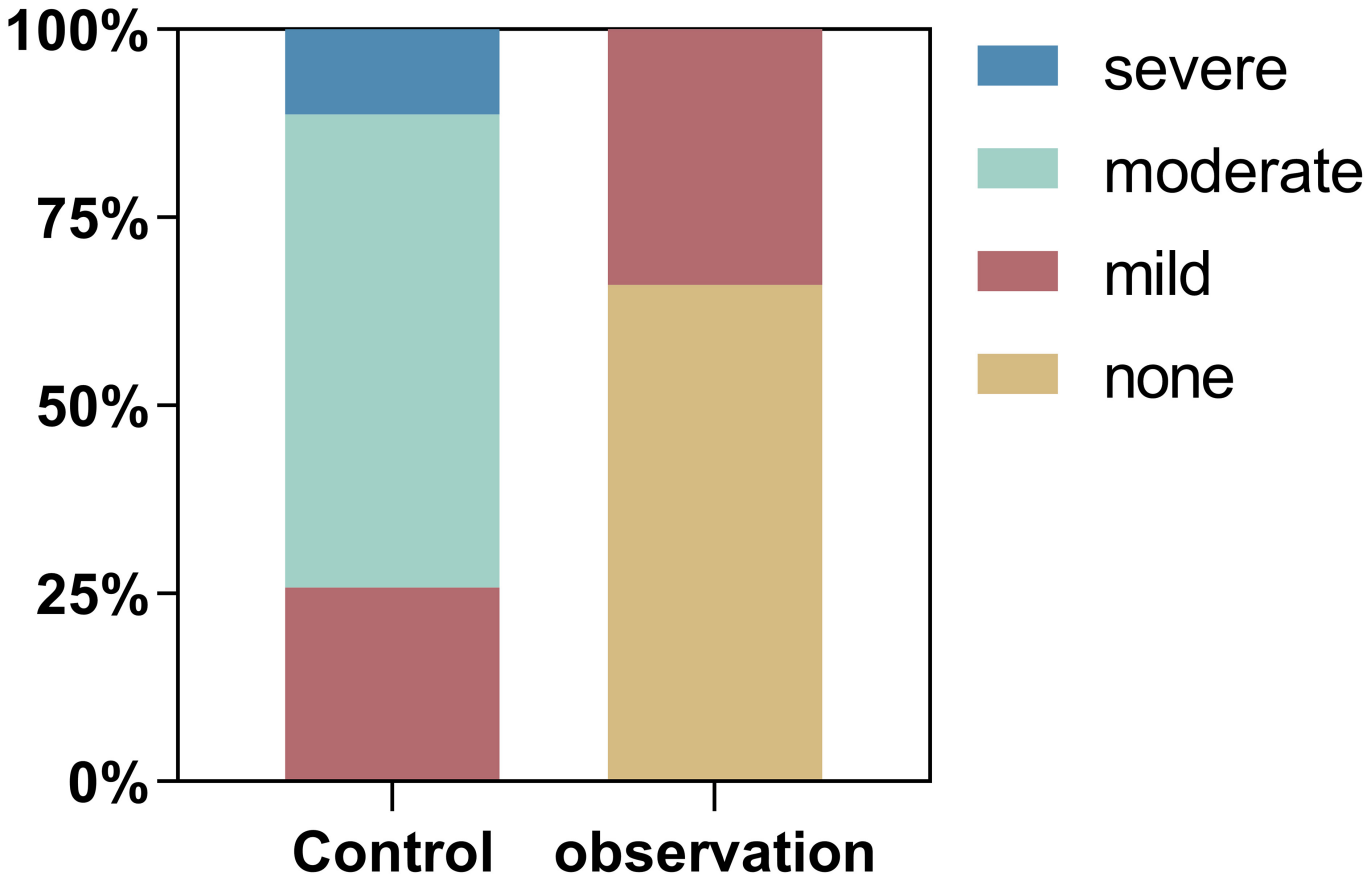
Proportion of patients reporting different levels of pain according to the Numerical Rating Scale (NRS) in the observation (FGM) and control (fingerprick) groups.

Compared to patients using traditional finger blood collection for monitoring blood glucose (control group), patients using FGM (observation group) experienced less pain during blood glucose monitoring (*P* < 0.05) according to NRS. In the observation group, 35 patients (66.04%) reported no pain and 18 patients (33.96%) reported mild pain. However, in the control group all patients reported pain. Of these, 16 (25.81%) patients reported mild pain, 39 (62.9%) patients reported severe pain and 7 (11.29%) patients reported severe pain (Figure 2).

### 3.3 Comparison of patient satisfaction with blood glucose monitoring devices between the two groups

As shown in Table 2, patients in the observation group were more satisfied with the blood glucose monitoring equipment than the control group according to GMSS. The median score for patients in the observation group was 70, compared to 47 for the control group. Statistically significant difference in satisfaction with blood glucose monitoring equipment between the two groups (*P* < 0.05). Informal qualitative feedback from patients in the FGM group frequently cited “reduced pain,” “convenience,” and “a feeling of better glucose control” as key reasons for their higher satisfaction.

**Table 2:** Comparison of patient satisfaction scores between groups.

**Group**	**Medium**	***Z***	***P-*value**
Control (*n* = 62)	47	9.213	0.000
Observation (*n* = 53)	70		

Satisfaction was assessed using the Glucose Monitoring System Satisfaction Survey (GMSS). Data are presented as median scores; the Mann–Whitney *U* test was used for comparison.

### 3.4 Comparison of abnormal glucose events and adverse events between the two groups

As shown in Table 3, abnormal glucose events (*P* = 0.027) and adverse events (*P* = 0.039) in the observation group differed from those in the control group. During the monitoring of all patients, 11 (20.8%) hypoglycemic events and 5 (9.4%) hyperglycemic events were monitored in the observation group; 4 (6.45%) hypoglycemic events and 10 (16.1%) hyperglycemic events were monitored in the control group. Hypoglycemic events could be more early detected in the observation group. Because finger blood sampling for blood glucose monitoring was usually done 2 h after a meal, the occurrence of hyperglycemia was monitored more often. In terms of adverse events, there were more adverse events in the control group (*P* = 0.039).

**Table 3:** Comparison of abnormal glucose events and adverse events between groups.

**Event**	**Control (*n* = 62)**	**Observation (*n* = 53)**	**c^2^**	***P-*value**
**Abnormal blood glucose event**				
Hypoglycemia	4	11	4.922	0.027
Hyperglycemia	10	5		
**Adverse events**				
Falling	1	1	8.356	0.039
Thirsty	7	4		
Incisional infection	2	0		

The Chi-square test was used for comparison.

## 4 Discussion

Glycaemia in patients after renal transplantation is a matter of great concern. Some patients require renal transplantation due to ESRD as a result of diabetes. However, hyperglycemia in the early post-transplant period often leads to renal graft failure and increased mortality (27, 28). Therefore, early monitoring of patients' blood glucose after kidney transplantation is essential to detect early hypoglycemic events for prevention. In this study, we compared the advantages and disadvantages of two methods of blood glucose monitoring after renal transplantation in order to obtain the best method of blood glucose monitoring.

Usually blood glucose is elevated in the perioperative period after kidney transplantation, which is consistent with our study. In this study, the incidence of both hyperglycemic and hypoglycemic events was 13.4%. The incidence of hypoglycemic events was higher than the 3.7% in the Chakkera' study (6), possibly due to the use of FGM to monitor patients' blood glucose around the clock. And most of the hypoglycemic events in our study were concentrated in the first week after surgery. The main reasons for this may be the prolonged pre-operative fasting, the patient's diet and the high metabolic state of the post-operative period causing frequent early hypoglycemia. Secondly, our patients took immunosuppressants at 6 am and 6 pm, and most of them would take longer between meals for the accuracy of the test, thus affecting their blood glucose somewhat. The incidence of hyperglycemic events was lower than the general incidence in the renal transplant population, again due to the use of FGM to monitor patients' blood glucose in real time and intervene in advance. However, early intervention for hyperglycemia inevitably creates the possibility of hypoglycemic events.

The use of FGM to monitor blood glucose is effective in reducing needle pain in patients. Pain and discomfort have been the focus of intensive research into blood glucose monitoring devices. Traditional blood glucose measurement methods are invasive and can cause discomfort to patients, thus affecting quality of life and motivation and compliance with blood glucose monitoring. The FGM used in this study is a non-invasive real-time blood glucose monitoring device that can significantly reduce the pain caused by blood collection (29). Of the patients who used FGM in this study, 35 (66%) patients reported no pain and 17 (34%) reported only mild pain. Our results are consistent with Bailey and Marcus' study (30, 31), where the pain and wellbeing of blood glucose monitoring using the FGM was much higher than that of finger glucose testing. However, the FGM is prone to skin discomfort and risk of infection when fixed to the skin, and there is a delay in blood glucose readings (30, 32). Therefore, the device needs further technical improvement, but it meets the criteria for clinical use. Our findings regarding reduced pain and improved satisfaction are consistent with the growing body of evidence supporting the use of FGM in various inpatient and outpatient settings. Recent studies have demonstrated that FGM can significantly improve patient adherence to glucose monitoring and quality of life by reducing the physical discomfort and logistical burden associated with frequent fingersticks (33).

The use of FGM to monitor blood glucose improves patient satisfaction with blood glucose monitoring devices. In this study, patients in the observation group were more satisfied with the blood glucose inter-monitoring device than the control group, and with a full score of 75 points, the median score in the observation group was 70 points compared to 47 points in the control group, and the difference in satisfaction with the blood glucose monitoring device between the two groups was statistically significant (*P* < 0.05). The main disadvantage of traditional glucose monitoring for patients who monitor their blood glucose for long periods of time is its inherent invasiveness, which is responsible for poor patient compliance. A study by Ward et al. showed that 50% of patients indicated that they would monitor their blood glucose levels occasionally, as needed (34). CGM devices, which allow automatic monitoring of glucose levels every few minutes, have been widely used for their comfort, usability, and accuracy (29, 31). A study by Polonsky et al. confirmed that FGM use increased patient satisfaction with the accuracy of the glucose monitoring device, and that patients were willing to use the FGM frequently and achieve better overall glycemic control (35). A study conducted by Halford et al. showed a significant reduction in the fear of hypoglycemia in patients when using an FGM to monitor their blood glucose (36). Thus, the use of FGM can reduce the burden on patients to some extent, and patients showed increased compliance with blood glucose monitoring and expressed greater satisfaction with the glucose monitoring device.

The use of FGM to monitor blood glucose can promptly detect abnormalities in the patient's blood glucose. Disorders of glucose metabolism are quite common in renal transplant patients in the short term due to post-transplant stress and the administration of immunosuppressive drugs. Ensuring that patients' blood sugar is stable is an important part of improving patient care and preventing abnormal blood sugar events (37, 38). The study found that the majority of all hypoglycemic events occurred during the patient's sleep and were unrecognized. 10 p.m. to 2 a.m. is a common time for hypoglycemia to occur at night (39). In this study, 11 cases (> 4 cases) of hypoglycemic events were detected in the observation group. This was due to the fact that the control group used FGM to monitor blood glucose, which monitors patients' blood glucose around the clock and allows a retrospective view of patients' blood glucose, so FGM identified more early morning hypoglycemic events.

It is worth noting that previous reports have indicated that FGM readings may be slightly lower than blood glucose during hypoglycemic episodes and may exhibit a physiological time lag during rapid glucose changes (40, 41) This characteristic might have contributed to the higher number of hypoglycemic events detected in the FGM group in our study. While this underscores the high sensitivity of FGM for hypoglycemia detection, clinicians should be aware of this potential discrepancy and consider confirmatory fingerstick tests in symptomatic patients or when hypoglycemia is suspected.

Finger blood glucose testing is not usually performed during sleep time, so finger blood glucose testing could miss many hypoglycemic events. In this study, 11 hypoglycemic events (>4) occurred in the observation group, while a much smaller number of hyperglycemic events (5) occurred. This may be due to the outcome of early intervention following glucose monitoring of patients using FGM. Furthermore, the use of corticosteroids such as prednisone in our immunosuppressive regimen is a well-known contributor to post-prandial hyperglycemia due to induced insulin resistance. Conversely, the aggressive management of this steroid-induced hyperglycemia with insulin, especially in the evening, may increase the risk of nocturnal hypoglycemia (42), particularly between 10:00 p.m. and 2:00 a.m., as observed in some of our cases. This highlights the critical need for balanced and cautious glycemic management in the early post-transplant period, where both hyperglycemia and hypoglycemia pose significant risks. It has also been suggested that strict and aggressive glycemic control may increase the risk of perioperative hypoglycemia (43). Therefore, there is a need to strengthen the monitoring and management of blood glucose in patients at times associated with a high incidence of abnormal blood glucose events.

The use of FGM to monitor blood glucose can reduce the incidence of glucose-related adverse events in patients. FGM provides an overview of trends in blood glucose fluctuations at all times and can minimize and avoid blood glucose related adverse events. Studies have shown that early post-operative hyperglycemia predicts post-operative glucose metabolism disorders (44), and that hyperglycemia occurring within 45 days in renal transplant patients increases the risk of CMV infection (14) and the development of proteinuria (45), increasing the length of hospital stay (46). In this study, there were no incisional infections in the observation group, 2 incisional infections and 7 cases of irritable thirst in the control group. FGM demonstrates significant benefits in reducing the incidence of adverse events.

This study explored the effect of FGM in early post-transplant patients. The results showed that the protocol was effective in reducing the painful pinprick sensation during glucose monitoring, detecting abnormal glucose events in a timely manner, reducing adverse events and improving patient satisfaction with the glucose monitoring equipment. While our study provides promising short-term results, larger multi-center randomized controlled trials with longer follow-up are needed to confirm the long-term benefits of FGM on hard endpoints such as PTDM incidence, graft survival, and patient mortality. Although all patients received a similar immunosuppressive regimen (TAC + MMF + prednisone), other potential confounders such as variations in steroid dosing, occurrence of infections, and other medications were not rigorously controlled for in this observational study. These factors could influence glucose metabolism and represent a limitation of our study design. Additionally, integrating FGM with clinical decision support systems represents a promising future direction to optimize glycemic management in complex post-transplant care.

## Data Availability

The raw data supporting the conclusions of this article will be made available by the authors, without undue reservation.
